# Thermal Comfort Properties of Biodegradable Hemp and Polylactide Fiber Knitted Fabrics

**DOI:** 10.3390/polym17070903

**Published:** 2025-03-27

**Authors:** Ingrida Padleckienė, Laimutė Stygienė, Sigitas Krauledas, Aušra Abraitienė, Audronė Sankauskaitė

**Affiliations:** Department of Textile Technologies, Center for Physical Sciences and Technology, Demokratu Str. 53, LT-48485 Kaunas, Lithuania; laimute.stygiene@ftmc.lt (L.S.); sigitas.krauledas@ftmc.lt (S.K.); ti@ftmc.lt (A.A.); audrone.sankauskaite@ftmc.lt (A.S.)

**Keywords:** thermoregulation, knit fabric, hemp, polylactide

## Abstract

According to the global strategy of Green course, the production of sustainable textiles using different biodegradable fibres has immense potential for the development of sustainable products. Using one of the most sustainable biobased pure hemp and polylactide fibers yarns, four new biodegradable three-layer weft knitted fabrics with good thermal comfort properties were developed. The inner layer (worn next to the skin) and the middle layer of the knits were formed of hydrophobic polylactide fibers, the outer layer of different amounts (36–55%) of hydrophilic natural hemp fibers. Biodegradable polylactide fiber yarns were used as a replacement for conventional petroleum-based synthetic fibers. Natural hemp fibers are one of the most sustainable fibers derived directly from *Cannabis sativa* L. plants. The properties of the knitted fabrics were analysed and compared under thermoregulatory-moisture management, thermal resistance, air and water vapour permeability-properties. The results showed that all newly developed knits are ascribed to ‘moisture management’ fabrics according to the summary grading of all indices of moisture management parameters. In addition, it was found that the highest overall moisture management capability is related to the quantity of natural hemp fiber composition in different knitting structures. Based on the overall moisture management capacity (OMMC) index and thermal resistance values of developed knitted fabrics, the performance levels for these materials contacting the skin and intended for the intermediate layer were determined.

## 1. Introduction

The most important functional purpose of knitted materials, used in high-performance physical activity or recreational products worn in close contact with the skin, is to ensure comfort while creating and supporting a stable and pleasant microclimate of the skin surface independent from the environmental conditions. Thermophysiological comfort is defined as the level how the body retains heat balance in the rest position or at various levels of activities [1,2]. Thermoregulation properties, where the most important is liquid moisture management, of textile materials are influenced by their structure and nature of the fibers, the treatments applied to the fabric, the amount of air between the fiber and the ability to transfer moisture from the body surface to the outer layer of the garment [3,4,5,6,7].

According to CEN/TR 16422 [8], depending on environmental conditions and levels of physical activity, the thermal regulation properties of textile materials are divided into three performance levels: A (very good), B (good), and C (medium).

Currently, most of the textile fabrics available on the market with good moisture management properties are made from natural fibers and polyester (PES) blends. To develop sustainable textiles and replace PES, biodegradable, compostable, and renewable biobased polylactide (PLA) fibers are suitable.

Sources from the scientific literature show that textile materials with good moisture management properties are usually composed of at least two layers. For the production of two-layer knitted fabrics with good/excellent moisture management properties, two main principles can be distinguished [9,10,11,12,13,14,15]: (1) the use of conventional or unique hydrophobic synthetic fibers that have a good capillary action for the inner layer and the outer layer that absorb wicked moisture and allow it to evaporate from hydrophilic fibers; (2) two-layer knits can be formed using only hydrophobic synthetic fibers of different geometric parameters or various cross sections in both fabric layers. Following the above principles, it is also possible to create three-layer knitted fabrics with good moisture transport properties. The three-layer fabric is very functional due to its multilayer construction since each individual layer can be formed from different fibers. The inner layer of these knits is intended for liquid absorption, the middle layer is intended for liquid transmission, and the outer layer is intended for effective moisture distribution and rapid evaporation to the environment.

Data related to investigations of thermal comfort properties of three-layered knitted fabrics, intended for use during increased physical activity for skin-contacting materials, can be found in the scientific literature. The article [3] presents the liquid moisture management properties of investigated three-layer weft knitted fabrics made using PES spun yarns and polyamide-textured filament yarns with different bioceramic additives containing FIR. The article [12] presents the studied liquid moisture management properties of three-layer weft knitted fabrics. The inner layer of developed fabrics was formed from polyester (PES) spun yarns, the middle layer was from PES textured yarns, and the outer layer was from wool, bamboo viscose, and cotton/PES spun yarns. Researchers [15] studied thermal comfort properties of tri-layer knitted fabrics made from different combinations of microdenier filament polyester, staple fiber polyester, and cotton spun yarns, also polypropylene filament yarns. In the mentioned cases, knitted fabrics with the best thermoregulatory properties were selected. As can be seen, these fabrics are not biodegradable and their use/production is not in line with the Green Deal strategy. To develop three-layer biodegradable weft-knitted fabrics and to ensure their good thermoregulatory properties, two of the most important tasks can be distinguished: properly selecting biodegradable hydrophobic/hydrophilic fibers/yarns and correctly designing the layered knitted structure.

The main goal of this research was to exploit the unique properties of biobased pure hemp and polylactide fiber yarns to design the three-layer structure of weft knits in order to develop biodegradable knitted fabrics with good thermal comfort properties.

It should be emphasised that no studies have been found in the scientific literature investigating the comfort properties of three-layer biodegradable knitted structures. On the market or in the scientific literature there are no analogues of the knitted fabric structure developed in this study, designed by combining pure hemp and polylactide fiber yarns. The listed arguments confirm the novelty of this research.

**Figure 1:**
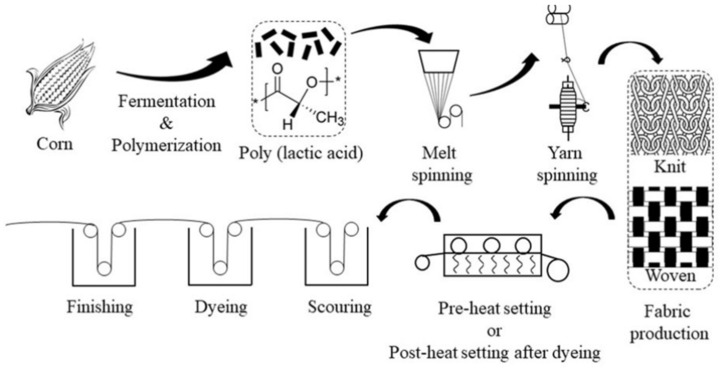
The production and processing stages of PLA textiles. Reprinted with permission from the study [18]. Copyright © 2020 by (Text. Res. J.). Reprinted by Permission of Sage Publications. *—a fragment of a polylactic acid monomer in the production and processing stages of PLA textiles.

PLA fibers derived from renewable plant sources, such as corn, are increasingly considered a sustainable alternative to petroleum-based synthetic fibers [16]. PLA is the only synthetic fiber produced from rapidly renewable resources and is a sustainable alternative to petroleum-derived fibers [17]. It is produced by fermenting corn starch to produce lactic acid, a major component of PLA. This process reduces dependence on non-renewable resources and makes PLA fibers a sustainable alternative to traditional fibers such as polyester [17]. Therefore, PLA fiber is considered a substitute for polyester fiber. PLA fibers combine the characteristics of synthetic and natural fibers, offering elasticity, UV resistance, and prevention of bacterial growth, making them suitable for various textile applications [18]. The Production and processing stages of PLA textiles are shown in Figure 1 [18].

**Figure 2:**
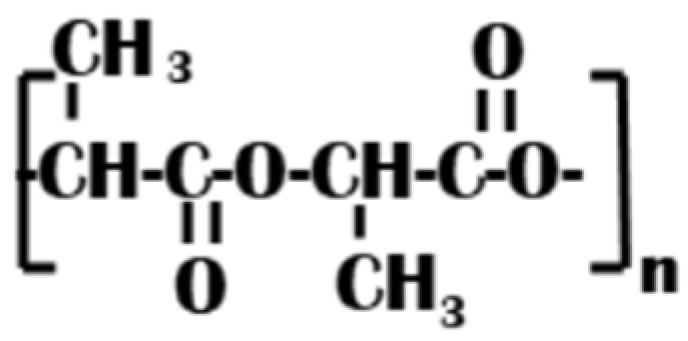
PLA structural formula.

PLA is one of the most promising polymers and is properly called the ‘polymer of the 21st century’. It is the only one, synthesized on a greater scale that is concurrently: biocompatible, biodegradable, and biobased [19]. PLA is an aliphatic polyester (Figure 2), primarily produced by industrial polycondensation of lactic acid (2-hydroxypropionic acid) and/or ring-opening polymerization of lactide. Lactic acid has two stereo-isomers, namely L-lactic acid (L-LA) and D-lactic acid (D-LA). Melt processing is the main technique used for the mass production of PLA products for the medical, textile, plasticulture, and packaging, and usually only high Mw PLA has a major commercial value in these industries [20]. Researchers [21] investigated PLA fabrics supplied by NatureWorks (USA) containing 2% D-LA and having an average molecular weight (Mw) of 58 kDa.

Although PLA fibers are hydrophobic (moisture regain 0.4–0.6%; water absorption 0.5%) in nature, due to the presence of unique multichannel cross sections, fibers are found to be more hydrophilic than polyester (moisture regain 0.2–0.4%; water absorption 0.3%) and lead to improving moisture transfer [18,22]. These properties are important for active wear and sportswear and therefore improve the comfort and wearability of the garment. As a result of their soft nature, PLA fibers are ideal for garments that come in direct contact with the skin. In contrast to polyester fibers, it does not accumulate static electricity. It is also non-irritating and minimizes the chances of having allergic reactions [17].

In addition to being energy efficient, PLA fibers are biodegradable under special composting conditions, reducing their environmental footprint when disposed of. PLA fibers are characterized by their ability to convert to nontoxic components (e.g., carbon dioxide and water) in 1 to 3 months under suitable conditions [17]. This biodegradability is a key factor in the reduction of textile waste, especially in an era where synthetic fibers contribute to significant environmental pollution. PLA fibers can also be recycled or reused, and they reduce overall waste.

Hemp fiber is considered one of the most sustainable and fashionable fibers, as it is directly obtained from *Cannabis sativa* L. plants, one of the oldest cultivated on the Earth [23,24,25]. Therefore, the development of functional textile materials containing eco-friendly hemp fibers is currently a priority goal. Natural hemp fiber is like flax fiber, but in terms of ecological benefits, hemp fiber is superior to many other plant-based cellulose fibers because it does not require herbicides or pesticides for cultivation [26]. It also consumes about three times less water than cotton [27]. That is why the highly sustainable hemp fiber often referred to as “new cotton” can successfully replace the cotton fiber currently used in the clothing industry.

Efficient photosynthesis during the growing process effectively reduces the emission of greenhouse gases, such as carbon dioxide CO_2_, into the atmosphere. Depending on the variety, one hectare of hemp plants can absorb approximately 10–22 tons of CO_2_ from the atmosphere during one growing season, improving air quality and thermal balance and having a positive impact on the environment [24,28]. Meanwhile, flax can absorb 4 to 12 times less CO_2_ than hemp (that is, 3.7 tonnes per hectare) [29].

The cultivation of hemp effectively improves soil quality due to its long root structure, which promotes soil oxygenation and natural leaf composting, which regenerates vital soil elements enriched with nitrogen [30]. Furthermore, one of the important properties of hemp is phytoremediation, during which toxic heavy metals (such as cadmium, lead, nickel, etc.) are absorbed and removed from the soil [26,30,31]. Therefore, by using hemp fibers as a raw material for clothing, this fact requires compliance with higher industry standards: the choice of the right land and seeds, as well as organic hemp fibers certification. Fiber hemp plants are known for their extraordinary robustness and, acting as natural herbicides, they can suppress other less valuable plants or weeds, thus reducing the need for crop rotation [24,32]. Hemp can be planted repeatedly on the same land and is often used as a rotation crop to heal the soil between growing other crops [33]. The height of cultivated hemp plants is approximately 3–3.5 m, but they can grow up to 6 m [34,35,36]. Hemp is a tall annual crop and can be grown in a short harvest period (70–90 days). Due to their high height and fast growth cycle, hemp plants produce 250% more fiber than cotton and 600% more fiber than flax on the same land and have the highest yield per acre of any natural fiber [33]. Hemp fiber products can be composted under soil conditions.

Hemp fiber is valued for its unique natural properties such as low density (1.43–1.48 g/cm^3^), long fiber length (up to 55 mm), high strength (specific strength ~ 0.45 N/tex), stiffness (50–70 GPa), durability, resistance to environmental factors (cold, heat, moisture, including salty sea water), molds and moths [35,36,37,38,39,40]. In the literature, hemp fiber has been reported to be longer than flax and cotton and therefore stronger (up to 8 times) than flax, and the lifetime of hemp is one of the longest and most durable of all natural fibers [41]. The hemp fiber or yarns are rougher than flax because hemp has more remnants of lignin than flax fiber [42]. The main components of hemp fibers are cellulose and hemicellulose. Hemp fiber consists of microfibrils arranged in hierarchical structures and embedded between lignin and hemicellulose. The middle layer of elementary fiber is formed by microfibrils that form a long cellulose chain [43]. Because the moisture absorptivity of hemp fiber is mainly influenced by its hydrophilic cellulose, the fiber can absorb a great deal of moisture, up to 30 percent of its own weight, and quickly wick away again. At 20 °C, it absorbs 12% moisture under 65% relative humidity (RH) and 30% under 95% RH. These values are higher than cotton and linen [44,45]. Due to its very high hydrophilicity, hemp fiber is easy to dye with intense colours [46,47]. Additionally, very valuable properties of this fiber are its resistance to UV radiation (it does not fade or disintegrate in sunlight), antibacterial properties, the ability not to absorb various unpleasant odours [48], anti-allergenic, aseptic, and antistatic effects [49,50]. Elementary hemp fiber typically shows a round polygonal outer shape with a narrow, round, or oval central cavity in the fiber, that is, the hemp fiber structure is hollow with micro-air chambers [51]. Textiles made of hemp fiber are highly breathable, have good thermal conductivity [37], and provide the wearer with excellent thermoregulation properties (due to the hollow fiber structure, hemp fiber is an effective insulator, therefore, it naturally warms in the cold and cools in hot weather) [37,52].

This change toward PLA and hemp could play an essential role in addressing the environmental problems of the textile industry, which is one of the world’s largest polluters.

## 2. Materials and Methods

For the design and development of multilayer knitted fabrics with good thermoregulation properties, the following natural and other biobased biodegradable fiber yarns were selected:

- Hemp (HA) spun yarns (raw white) 27.8 tex (manufacturer—MAEKO S.r.l., Milan, Italy);
- HA spun yarns (raw white and dyed) 19.2 tex (manufacturer—Linificio e Canapificio Nazionale S.r.l., Villa d’Almè, Bergamo, Italy);
- Polylactide (PLA) spun yarns (raw white) 19.7 tex (manufacturer—PT Kewalram Indonesia, Kab. Sumedang, Bandung, Indonesia);
- PLA textured yarns (raw white) 16.7 tex/36 filaments (manufacturer—Indorama Ventures Fibers Germany GmbH, Bobingen, Germany).

27.8 tex and 19.2 tex were made from bleached, 100% long hemp fiber. In addition, two-ply twisted HA yarns of total (calculated) linear density 38.4 tex were manufactured using two single (raw white with dyed) HA spun yarns 19.2 tex. Twisting was performed with a ring twisting machine PL-31 (twist direction ‘S’, number of twists 160 m^−1^). Furthermore, two types of biodegradable polylactide fiber yarns (spun and textured filament) were selected to produce designed knitted fabrics.

**Figure 3:**
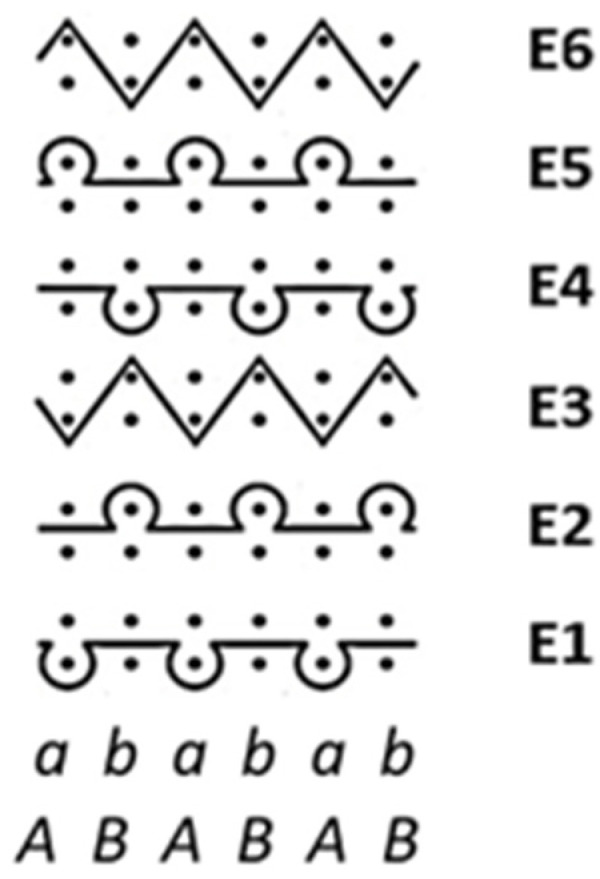
The combined pattern scheme of investigated three-layer weft knitted fabrics, where: E1–E6—the pattern feeds; A, B—the cylinder needles, forming the outer layer; a, b—the disc needles, forming the inner layer (knitted and tuck loops are marked according to the standard EN ISO 4921 [53]).

For investigation, three-layer knitted fabrics were knitted in a combined pattern on the gauge 20E interlock circular knitting machine and finished using steaming shrinkage, washing and impregnation with non-ionic softening agent in padding squeezing machine at 120 ± 5 °C temperature with overfeed 10%. The schematic pattern view of the designed knitting structures is presented in Figure 3, and the main characteristics of the knitted fabrics tested are presented in Table 1.

**Figure 4:**
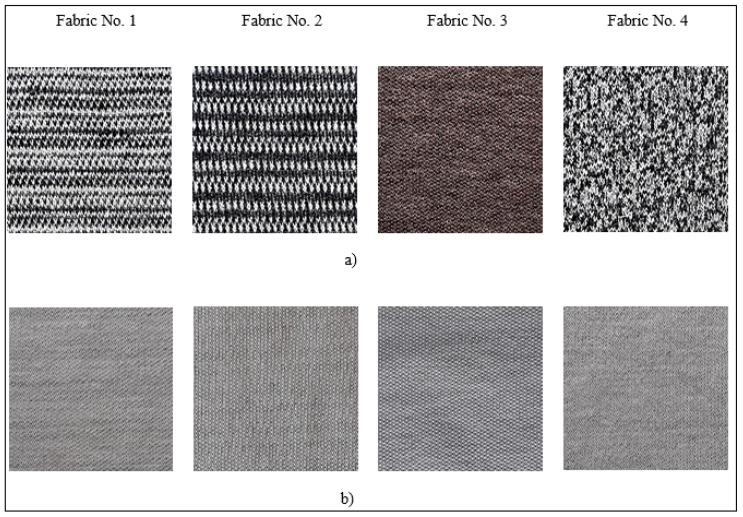
The view of the designed weft knitted fabrics: (**a**) the front side (outer) layer; (**b**) the back side (inner) layer.

The inner layer worn next to the human body or above the skin contact as the second (intermediate) layer of all knitted fabrics was formed from very thin (1.3 dtex) PLA fiber spun yarns 19.7 tex and therefore has a very soft and gentle grip. 16.7 tex textured filament yarns made from 4.6 dtex PLA fibers are incorporated into the middle layer of knitted fabrics. For providing good moisture management properties, the outer layer of the knits was made up of different amounts of hydrophilic hemp fiber spun yarns. Figure 4 presents the general view of the designed knitted fabrics.

The number of stitches was calculated according to the EN 14971 [54] standard and the mass per unit area was determined according to the EN 12127 [55] standard. The mean loop length of the investigated knitted fabrics was determined from the expression of the theoretical area density [56]:

*l_m_* = *A*·*B*·*M/T*, (1)

where: *l_m_* is the mean loop length of the knitted fabric in mm; *M* is the mass per unit area of the knitted fabric in g/m^2^; *A* is the wale spacing of the knitted fabric in mm; *B* is the course spacing of the knitted fabric in mm; *T* is the linear density of the yarns in tex.

For the determination of the tightness factor *TF* the formula [57] was used:

*TF* = *T*^1/2^/*l_m_*. (2)

The calculated coefficient of variation of the structural parameters of the investigated knitted fabrics was less than 3%.

**Figure 5:**
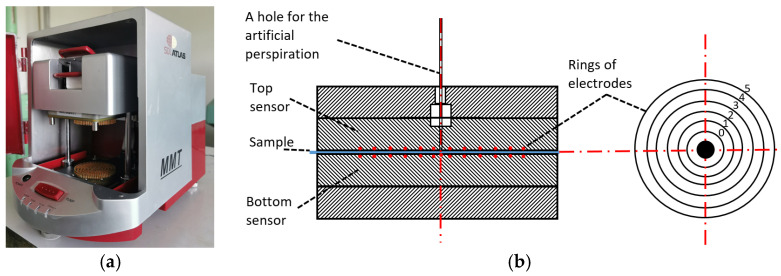
General view (**a**) and principal scheme (**b**) of the device for the determination of the moisture management properties.

Moisture management properties parameters were determined according to the AATCC test method 195 [58]. All materials were washed (according to EN ISO 6330 [59], washing procedure 4 N (40 °C), drying procedure A—flat dry) and conditioned for 24 h prior to testing. The moisture management properties of textiles were evaluated by placing the sample between two horizontal electrical sensors (the scheme of the device MMT M290 (SDL Atlas, Rock Hill, South Carolina, USA), shown in Figure 5). The main parameters used to describe moisture management properties and their levels are given in the standard AATCC 195.

**Figure 6:**
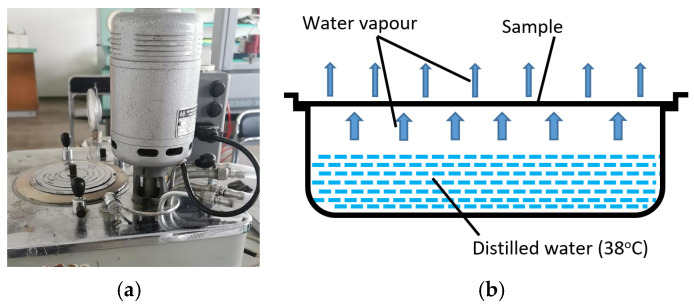
General view (**a**) and principal scheme (**b**) of the water vapour permeability test (“cup method”).

The original “cup method” was used to determine the water vapour permeability (“breathing”) of fabrics. The measure of this characteristic is the amount of water evaporated from the covered vessel in 6 h. A schematic view of the water vapour permeability testing device is presented in Figure 6.

The specimens for water vapour permeability test were prepared as follows: 3 specimens of 115 mm diameter were cut from various places of the fabric and weighed, then conditioned in a standard climate for 24 h. A glass container with 500 mL of distilled water was placed in the thermostat. The specimen was sealed over the vessel and the test duration was set at 6 h. The ambient temperature during the tests was 22 °C, the temperature of the distilled water 38 °C. After 6 h, the specimen was weighted and the moisture content in m_d_ was calculated.

m_d_ = m_2_ − m_1_, (3)

where: m_1_, m_2_—the mass of the specimen before and after the test, respectively, g.

The quantity of water m_v_ evaporated within 6 h from the glass container was calculated as follows.

m_v_ = m_3_ − m_4_, (4)

where: m_3_, m_4_—the mass of water in the glass container before and after the test, respectively, g.

The water vapour permeability WVP (mg/m^2^·s) was calculated using the formula:

WVP = ((m_v_ − m_d_)/q)/21.6 (5)

where: q—the area of the specimen through which the water vapour evaporated.

The coefficients of variation of the studied water vapour permeability did not exceed 8%.

**Figure 7:**
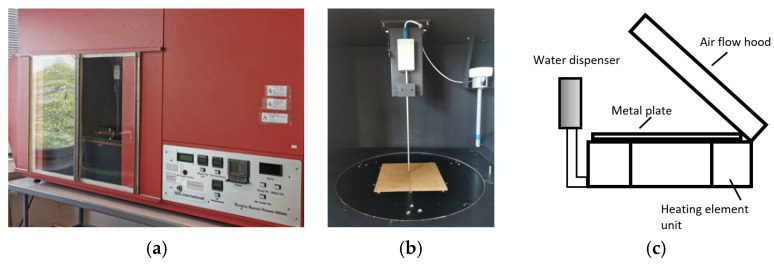
Device for the determination of the thermal resistance of fabrics: general view (**a**), porous plate (**b**) scheme (**c**).

The thermal resistance of knitted textiles was determined using a standardized procedure EN ISO 11092 [60], using a Sweating Guarded Hot Plate M259B device (SDL International Ltd., Radcliffe, Manchester, UK) (see Figure 7), which simulates human skin, the basic part of which is a porous metal plate, which heats up to 35 °C, that is, the temperature of the human body. The metal plate is approximately 3 mm thick and has an area of 0.04 m^2^. The coefficients of variation of the thermal resistance measurements did not exceed 5%.

**Figure 8:**
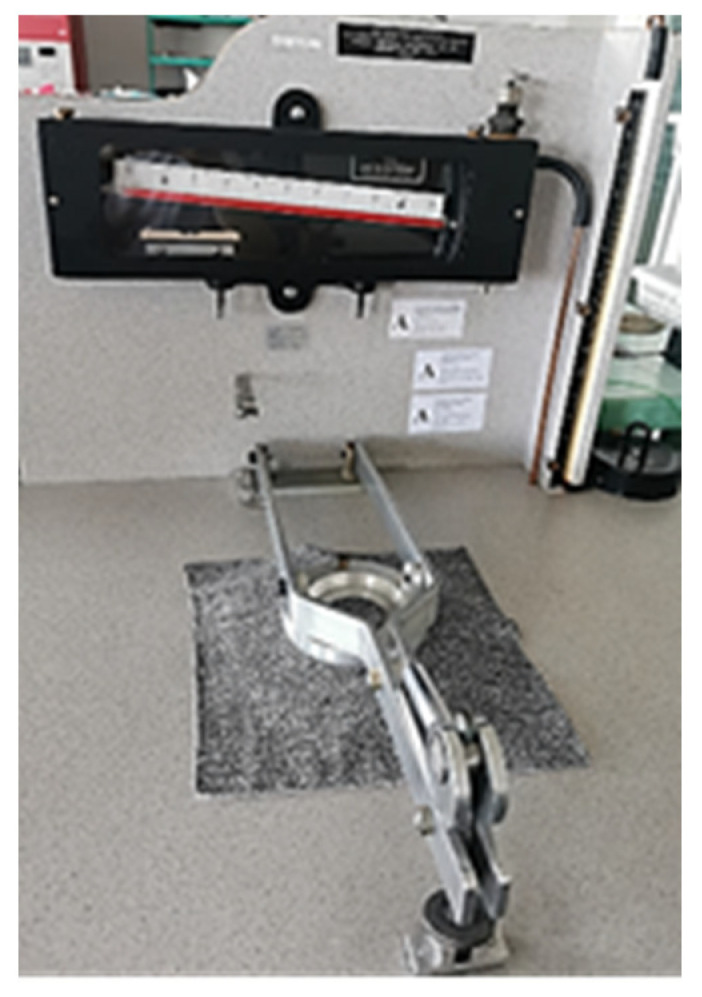
Frazier low differential pressure air permeability tester FAP-LP.

Air permeability tests were carried out with the Frazier low differential pressure apparatus FAP-1034-LP (Frazier Precision Instrument Company Inc. Tm., Hagerstown, Maryland, USA) according to the EN ISO 9237 standard [61], with a pressure drop of 100 Pa and a test surface area of 20 cm^2^ (Figure 8). The coefficients of variation of the measurements were approximately 7%.

The results were substantiated by fundamental statistical analysis using Microsoft Excel software. For each fabric sample, three measurements were performed, maintaining a significance level of α = 0.05. Statistical data, including average, maximum and minimum values; standard deviation (SD); coefficient of variation (CV); and the 95% confidence interval (CI), were calculated for each measurement per sample.

## 3. Results and Discussion

The moisture management capabilities of four different knitted fabrics, as detailed in Table 1, are investigated in this research. In this research the analyses of wetting time, absorption rate, the largest wetted radius, spread speed, and overall moisture management capacity were performed. The grading system helped quantify the performance and compare fabrics. The correlations between various parameters were investigated, and dependencies were shown.

In Table 2, we categorize and summarize the moisture management properties based on various indices. Summary statistical data for each MMT measurement per sample are presented in Table 3.

**Figure 9:**
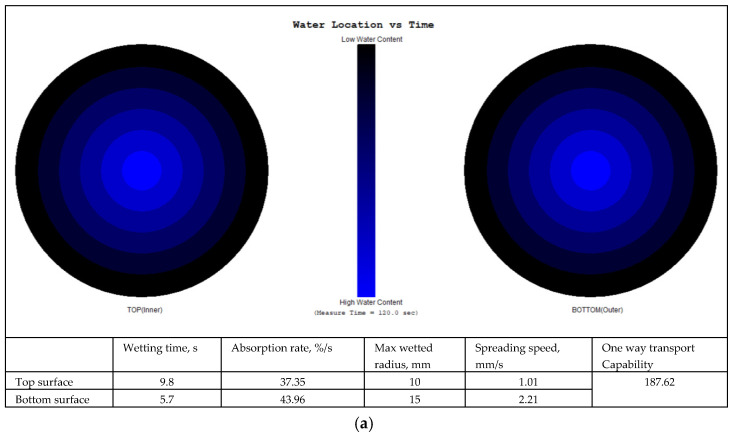

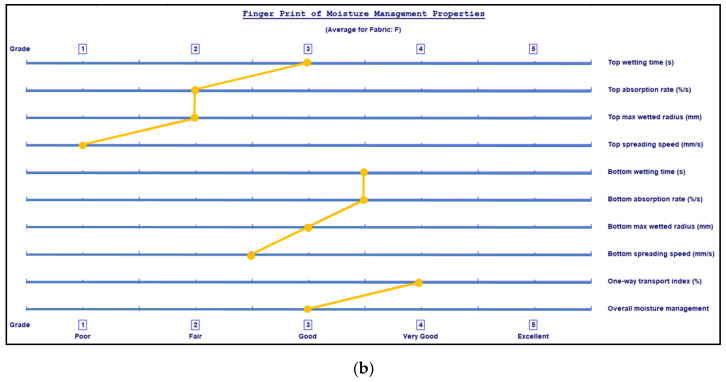
Diagram of the final liquid water spread location for the water content (water is marked by a blue colour) on the fabric No. 1 two surfaces (**a**) and the fingerprint of the moisture management properties with grading of the MMT indices (**b**).

**Figure 10:**
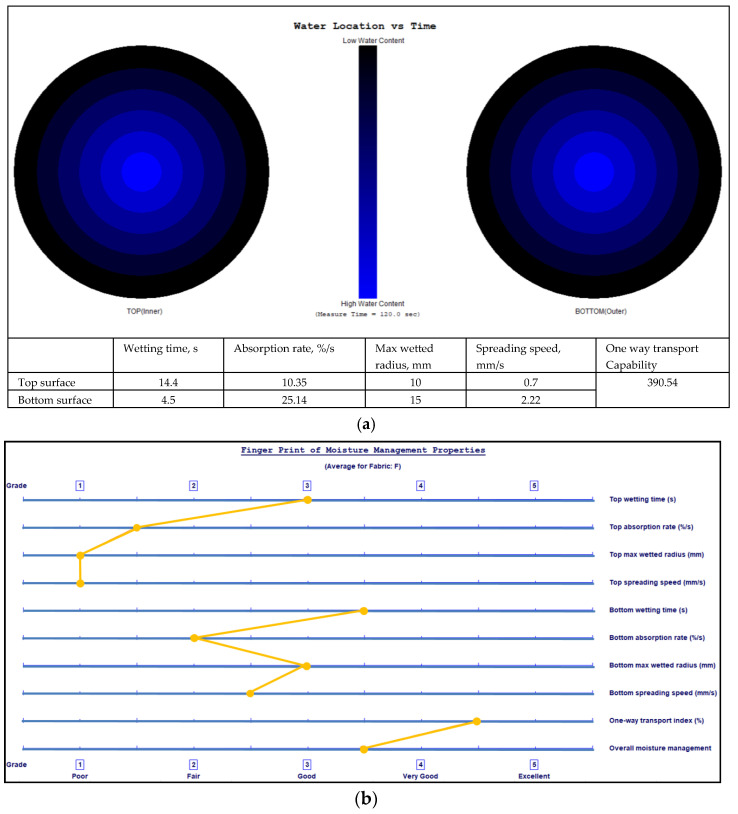
Diagram of the final liquid water spread location for the water content (water is marked by a blue colour) on the fabric No. 2 two surfaces (**a**) and the fingerprint of the moisture management properties with grading of the MMT indices (**b**).

**Figure 11:**
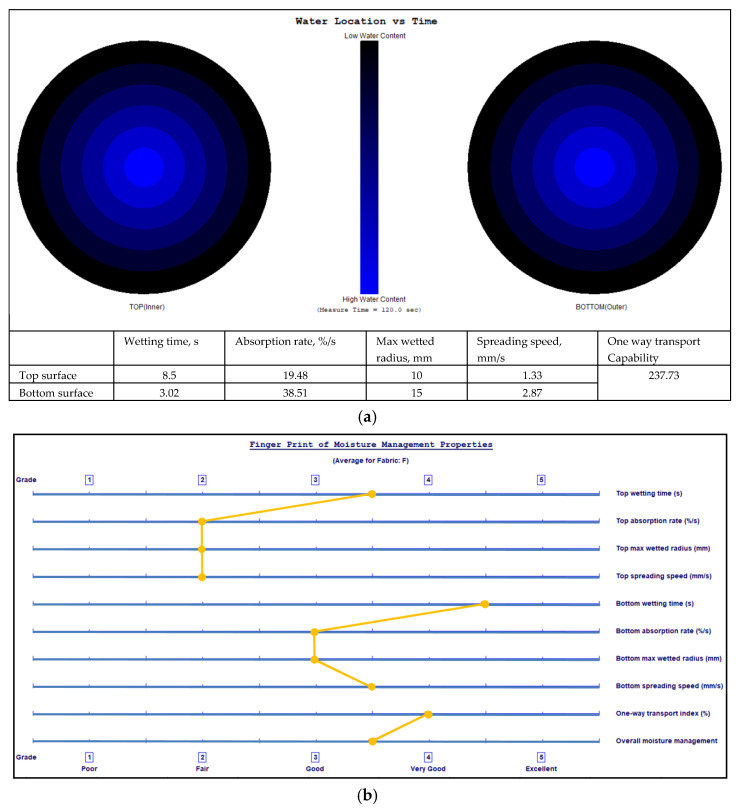
Diagram of the final liquid water spread location for the water content (water is marked by a blue colour) on the fabric No. 3 two surfaces (**a**) and the fingerprint of the moisture management properties with grading of the MMT indices (**b**).

**Figure 12:**
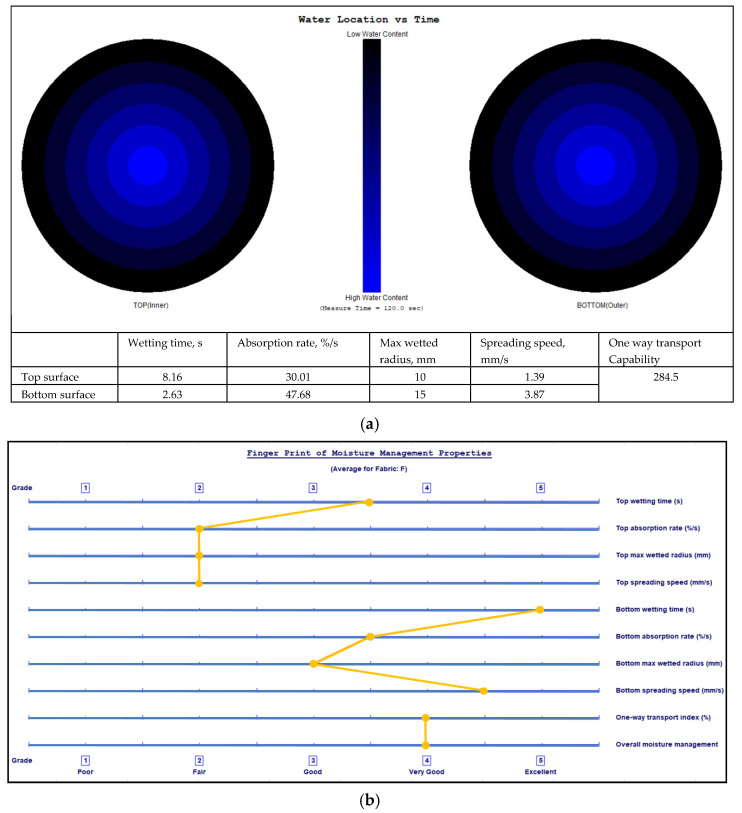
Diagram of the final liquid water spread location for the water content (water is marked by a blue colour) on the fabric No. 4 two surfaces (**a**) and the fingerprint of the moisture management properties with grading of the MMT indices (**b**).

The final location of the liquid water spread on the two fabric surfaces and fingerprints of the moisture management properties with grading of the MMT indices are shown in Figure 9, Figure 10, Figure 11 and Figure 12 for fabrics No. 1–4, respectively.

The wetting time is the time in seconds when the top and bottom surfaces of the fabric begin to wet after the test is started. As is seen from Table 2, Fabric No. 1, according to recorded time, exhibits a moderate absorption rate. Fabric No. 2 shows a prolonged wetting time on the top surface (medium, 3), and a comparatively lower moisture absorption efficiency than Fabric No. 1 on the top side. However, it shows better performance on the bottom side. Fabric No. 3 wetting time on the bottom surface is classified as very fast—5, with superior moisture absorption capabilities. Fabric No. 4 shows similar results as Fabric No. 3, that is, superior moisture management on the bottom surface.

The absorption rate describes the average speed of liquid moisture absorption for the top and bottom surfaces of the fabric during the initial change of water content during a test. The absorption rate of fabric No. 1 indicates a moderate capability for moisture absorption, particularly on the bottom side (absorption index—medium, 3). Fabric No. 2 shows worse performance on the bottom surface absorption (absorption index—slow, 2). Fabrics No. 3 and No. 4 show similar absorption rates on the top and on the bottom (absorption index—medium, 3) surfaces. Comparatively, fabric No. 1 has the highest absorption rate on the bottom surface, indicating that it effectively wicks moisture away from the skin.

The maximum wetted radius indicates the accumulated rate of surface wetting from the centre of the specimen, where the test solution is dropped to the maximum wetted radius. This radius for all fabrics shows a medium moisture spread on the bottom side and a small or no wetting on the top side. These measurements indicate that the inner layer of all fabrics, formed from PLA fibers, has a very small wetting area, i.e., it effectively absorbs liquid moisture and transfers it to the outer layer.

The speed of moisture spread is another essential factor in fabric evaluation, indicating the accumulated rate of surface wetting from the centre of the specimen, where the test solution is dropped to the maximum wetted radius. Fabric No. 1 shows a very slow spreading speed on the top surface and a slow on the bottom surface. Fabric No. 2 showed better moisture movement than fabric No. 1. Fabrics No. 3 and No. 4 exhibit the best parameters of spreading speed (index—fast, 4) on the bottom surface formed from pure hemp fibers. Fabrics with higher spread speeds are better at managing sweat during physical exertion, thus improving comfort for the wearer.

Accumulative One-Way Transport Capability is a critical metric for evaluating the fabric’s ability to transport moisture away from the skin. It is the total measure of the difference in moisture accumulated on the fabric’s inner and outer surfaces. All investigated fabrics having AOTC values from 267.08% to 385.29% indicate very good (index 4) liquid moisture transport. AOTC values reflect the overall moisture management functionality of the fabrics, with higher values indicating better performance in keeping the wearer dry.

The Overall Moisture Management Capability values are important for summarizing the overall performance of each fabric in moisture management. Fabric No. 1 has an OMMC score of 0.5359, categorized as good—3. Fabric No. 2 follows closely with a score of 0.6026, indicating a very good—4 performance. Fabric No. 3 highlights an OMMC of 0.621, also rated as very good—4, while fabric No. 4 achieves an OMMC of 0.7175, making it the standout performer with a classification of very good—4.

The results indicate that all newly developed knits have good or very good moisture management. Knits made with a higher content of hemp fiber absorbed an increased amount of liquid moisture compared to knits with a lower fiber content. The highest OMMC value (0.7175) was achieved with a fabric containing 55% hemp fiber, demonstrating a clear positive correlation between fiber content and moisture management capability. This finding is consistent with the previous literature [44,45,46,47] emphasizing the hydrophilic nature of pure hemp, which aids in moisture wicking.

**Figure 13:**
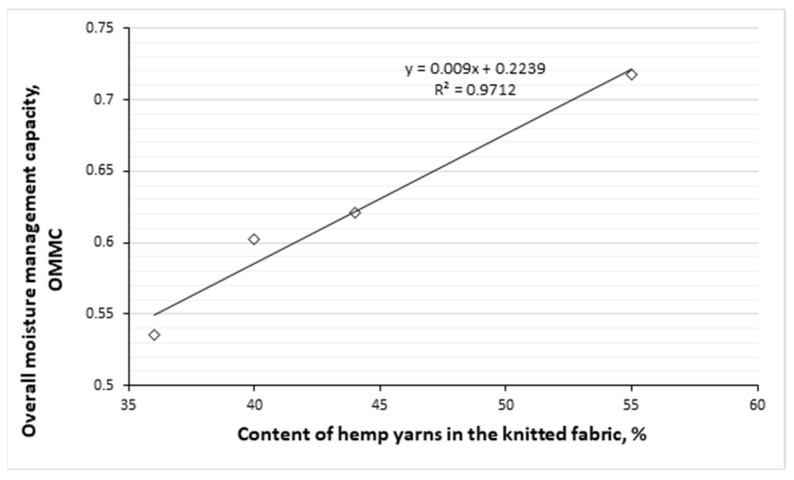
Correlation between overall moisture management capability and hemp fibers content in knitted fabrics.

As illustrated in Figure 13, there is a strong positive correlation between the content of hemp fibers in knitted fabrics and their OMMC. The regression equation with an R^2^ value of 0.9712 indicates that as the percentage of hemp fibers increases, the OMMC also increases, and the fabric’s ability to wick liquid moisture away from the skin improves. This correlation shows that the content of hemp fibers contributes significantly to the moisture-wicking properties of the fabric. This could also be explained by the fact that when the content of hemp fibers increases, the capillaries forming spaces between these fibres decreases. Hence, the narrower the capillaries, the greater the ability of the textile fabrics to wick moisture [12].

**Figure 14:**
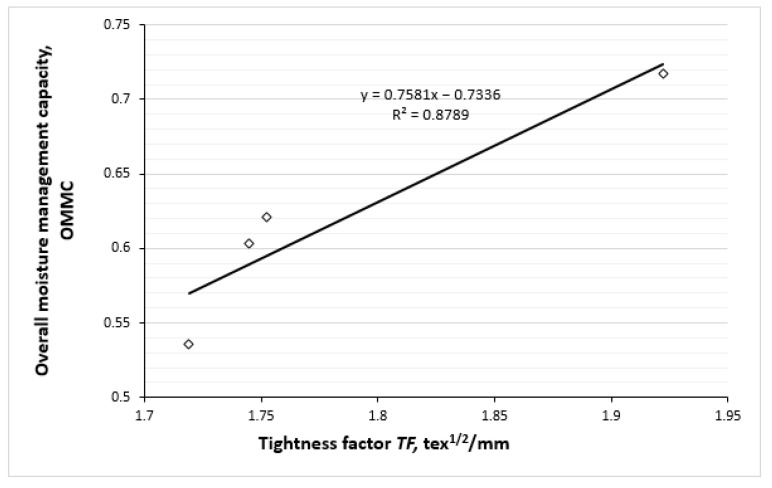
Correlation between overall moisture management capability and the tightness factor of knitted fabrics.

Figure 14 further emphasizes the correlation between the tightness factor of knitted fabrics and their overall moisture management capability. The regression equation with an R^2^ value of 0.8789 demonstrates that higher tightness factors correspond to better OMMC. A higher tightness factor indicates a denser fabric structure, which can enhance the fabric’s ability to manage moisture. This is particularly important for active wear, where moisture transfer is critical to maintaining comfort and thermoregulation.

**Figure 15:**
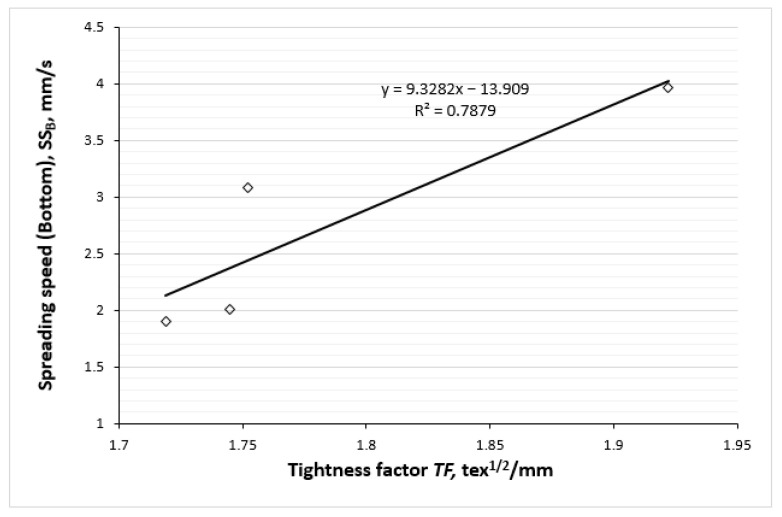
Correlation between spreading speed of liquid moisture on the outer surface of knits and their tightness factor.

The correlation between the spreading speed of liquid moisture on the outer surface of knitted fabrics and the tightness factor is illustrated in Figure 15. The regression equation with an R^2^ value of 0.7879 indicates a strong positive correlation. Fabrics with a higher tightness factor (i.e., having higher hemp fiber content in the outer layer of the knits) show a faster spreading speed of liquid moisture, which is beneficial for moisture management. This correlation reinforces the importance of optimizing the structure and composition of the layered fabric for better performance. A well-structured fabric that balances tightness and fiber content can optimize moisture management, thereby improving the user experience.

**Figure 16:**
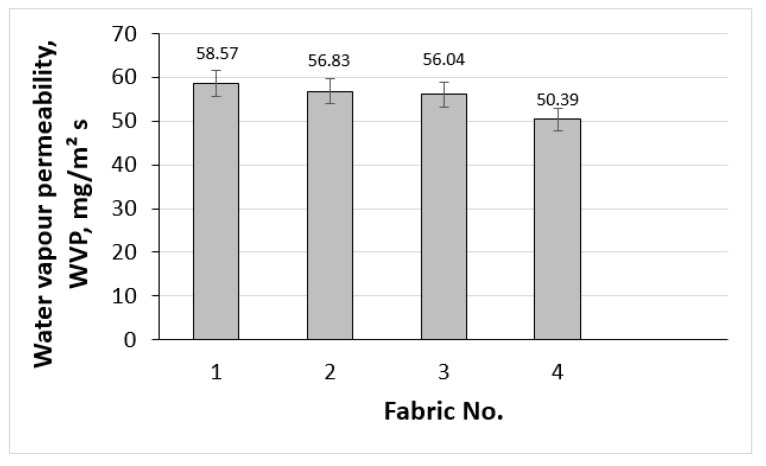
Water vapour permeability, WVP of investigated fabrics.

**Figure 17:**
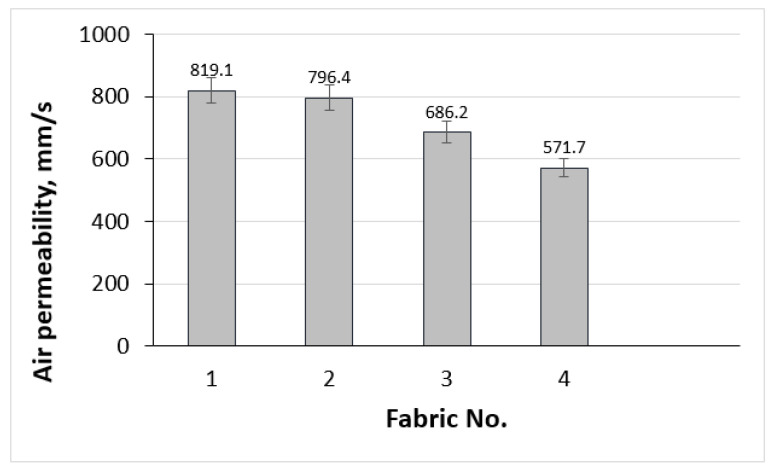
Air permeability of investigated fabrics.

The graphs showing water vapour and air permeabilities across different fabrics are presented in Figure 16 and Figure 17. Water vapour permeability reflects the fabric’s ability to allow moisture vapour to escape, which is critical to preventing the accumulation of sweat during physical activities. The WVP values for the fabrics range from 50.39 mg/m^2^ s for fabric No. 4 to 58.57 mg/m^2^ s for fabric No. 1 (see Figure 16). In particular, fabric No. 1 has the highest WVP showing very good fabric breathing properties ensuring comfort. A higher WVP shows that the fabric can effectively manage moisture in form of vapour without leading to a wet sensation on the skin. Analysing sources from the scientific literature [62,63], it can be seen that in this study the obtained values of the water vapour permeability provide a sufficiently good “breathability” function.

Air permeability is another significant factor that influences the thermoregulatory properties of fabrics. Air permeability values for the fabrics range from 571.7 mm/s to 819.1 mm/s (see Figure 17). Fabric No. 1 indicates excellent air circulation, facilitating better breathability, which is essential for comfort during physical activities. Higher air permeability allows excess heat and moisture to escape, contributing to a stable microclimate next to the skin. Fabric No. 4 still maintains good air permeability but has a tighter knit structure that may affect moisture management capabilities. The results obtained are consistent with the statements of other authors [63] that when TF increases, the spaces between loops, i.e., sizes of pores, through which the airflow permeates decreases. As a result, air permeability also decreases. Considering standard [8], materials can be assigned to the highest performance level by assessing air permeability if their air permeability is greater than 100 mm/s.

**Figure 18:**
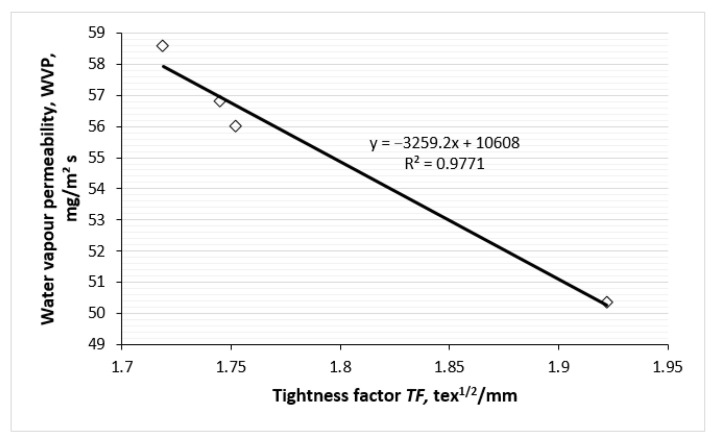
Correlation between the water vapour permeability of the knits and their tightness factor.

**Figure 19:**
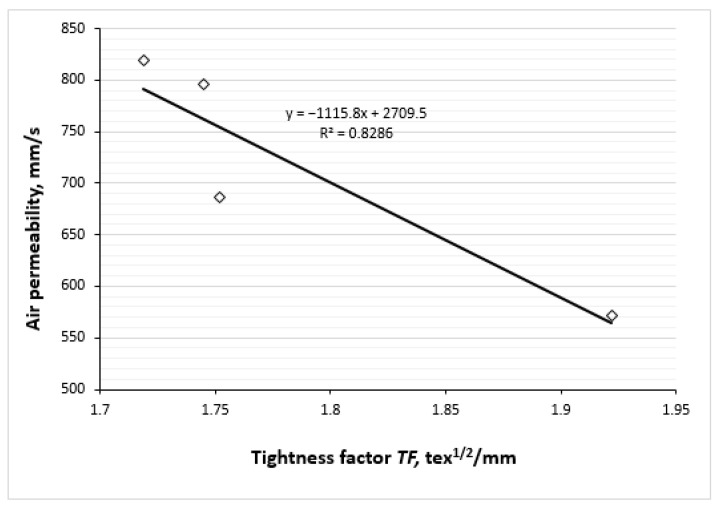
Correlation between air permeability of knits and their tightness factor.

The correlations between the tightness factor of the knitted fabrics and their water vapour and air permeabilities are presented in Figure 18 and Figure 19. As illustrated in Figure 18, there is a strong negative linear correlation between the permeability of the water vapours of the knits and their tightness factor. Figure 19 shows the correlation between the air permeability of the knits investigated and their tightness factor. It is generally accepted that the air permeability of a fabric depends on its porosity/tightness factor, which in turn influences its openness. In our case, as the tightness factor of knitted fabrics increases, the porosity decreases. Therefore, the air permeability of fabrics also decreases. As can be seen, between these variable parameters there is also a strong negative linear correlation. Both dependencies can be explained by the generally accepted fact that a denser knit structure has less space between the yarns and correspondingly lower porosity, and more permeable fabrics are obtained with looser structure.

**Figure 20:**
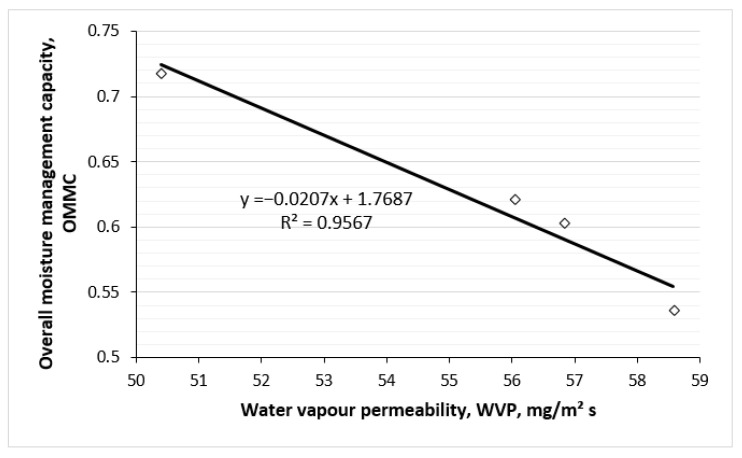
Correlation between overall moisture management capability and water vapour permeability of knitted fabrics.

**Figure 21:**
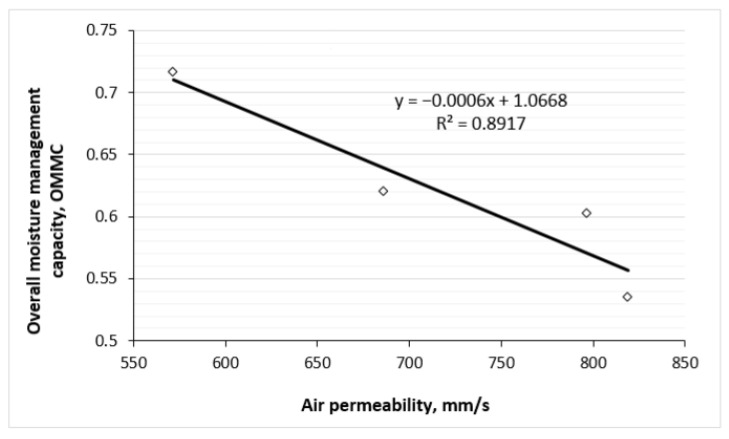
Correlation between overall moisture management capability and air permeability of knitted fabrics.

Figure 20 and Figure 21 show the correlations between overall moisture management capability and water vapour/air permeability of knitted fabrics. Strong negative linear correlations exist between the OMMC and WVP parameters also between the OMMC and the air permeability parameters. Higher values of OMMC correspond to lower values of water vapour and air permeability. This can be explained by the fact that OMMC increases with increasing TF, i.e., by the higher content of hemp in the outer layer of the knits, also increases. Meanwhile, by increasing TF (see Figure 18 and Figure 19), the parameters of water vapour and air permeabilities decrease.

**Figure 22:**
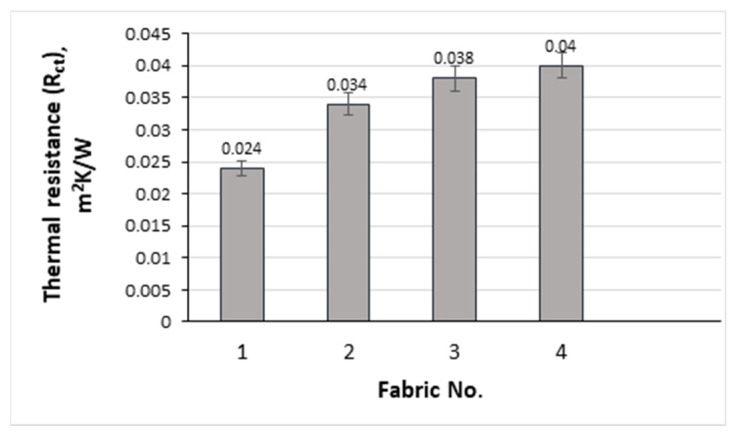
Thermal resistance of investigated fabrics.

In this study, the thermal resistance (R_ct_) values range from 0.024 m^2^K/W for fabric No. 1 to 0.040 m^2^K/W for fabric No. 3 (see Figure 22). A higher R_ct_ value indicates better thermal insulation, which is particularly beneficial in colder climates, where maintaining body heat is crucial. As expected [64], higher thermal resistance was obtained at higher TF and mass per unit area of the investigated knitted fabrics.

The results of statistical processing of the experimental data for each WVP, air permeability and R_ct_ measurement by knitted samples are presented in Table 4.

To choose the right level of each property and determine the specific intended use of the developed knits, the thermoregulatory properties were evaluated according to CEN/TR 16422. All main parameters that show the thermoregulation properties of the developed and investigated knitted fabrics and their performance levels are summarized in Table 5.

Analysing the data presented in the table, it can be seen that with respect to the standard CEN/TR 16422 the created three-layer knitted fabrics are suitable for skin contact materials worn in warm/cold climate conditions. According to the R_ct_ values, the knits correspond to the B (good), C (medium), and according to OMMC and their index value—to the B (good), A (very good) performance levels. These knits can be used in warm/cold climate as well as for second (intermediate) layer materials. According to the R_ct_ value investigated, knits correspond to the B (good), C (medium) and according to the OMMC and their index value to the A (very good) performance levels. It should be noted that all knits (except No. 1) according to OMMC and their index value corresponds to the highest (A) performance level intended for skin contact also for intermediate layer materials in both warm and cold climate conditions.

The limitation of standardized laboratory testing is not fully capturing real-world variations in humidity and temperature. To address this, it is appropriate for future research to include field tests in diverse climatic conditions to assess the fabric’s moisture management performance more accurately. Testing in controlled climate chambers simulating different temperatures and humidity levels would also provide valuable insights into the dynamic interaction between hemp-PLA fibers and environmental factors.

## 4. Conclusions

Biodegradable weft knitted fabrics with good thermal comfort properties were developed thanks to the synergy of a correctly designed three-layer knitted structure and the unique positive properties of selected pure hemp and PLA fibers yarns.

Following the summary of the grading obtained from nine moisture management indices, all knits developed were assigned to moisture management fabrics. The overall moisture management capability (OMMC) values of these fabrics were in the range of 0.5359 to 0.7175, and the OMMC was rated as good/very good. The analysis of moisture management parameters of the knits containing in the outer layer only natural hemp fiber has shown that the highest OMMC is directly related to the quantity of natural hemp in the knitted structure. 55% of hemp fiber in the knit fabric resulted in the highest OMMC value of 0.7175, which shows a very good moisture management performance. Between the content of hemp fiber and the OMMC values, a strong positive correlation was determined (R^2^ = 0.9712).

Strong positive correlations between OMMC and the tightness factor (TF), also between the spread speed of liquid moisture on the outer surface of the knits and TF were determined (R^2^ = 0.8789; R^2^ = 0.7879, respectively). Meanwhile, between OMMC and water vapour permeability (WVP) and between OMMC and air permeability parameters the strong negative correlations were obtained (R^2^ = 0.9567; R^2^ = 0.8917, respectively). Furthermore, strong negative correlations exist between the WVP and TF parameters (R^2^ = 0.9771) and between the air permeability and the TF parameters (R^2^ = 0.8286).

The results of the water vapour and air permeability parameters showed that newly developed fabrics are sufficiently breathable and permeable to air and may be used to be worn next to the skin and as an intermediate layer of clothing. In reference to the standard CEN/TR 16422, according to the OMMC index and thermal resistance values, it has been established that all knitted fabrics are suitable for use to be worn next to the skin and as an intermediate layer of clothing in warm and cold climate conditions during increased physical or recreational activity. All knits (except No. 1, performance level B) according to OMMC values correspond to the highest (A) performance level intended for skin contact also for intermediate layer materials in warm/cold weather.

This study highlights the importance of the three-layer biodegradable knitted fabric structure, including the tightness factor and the layer design, in determining moisture management and thermal comfort. The results obtained from this study are practically applicable. Three-layer biodegradable knitted fabrics with good thermal comfort properties in case of increased physical activity of humans can be used for sports, leisure, and recreational clothing.

The findings of this research have significant implications for the design and development of sustainable textiles. As the demand for high-performance and eco-friendly fabrics increases, the transition to biodegradable materials such as pure hemp and polylactide aligns with global sustainability goals. By focusing on materials that minimize environmental impact while providing excellent performance, the textile industry can contribute to a more sustainable future.

## Figures and Tables

**Table 1:** The main characteristics of knitted fabrics tested.

Fabric No.	Type of Separate Layers	Type of Yarns, Linear Density, tex	Arrangement of Yarns in Repeat of Knitted Fabric Pattern: Pattern Feeds (See Figure 3)	Content of Yarn in the Knitted Fabric, %	Number of Stitches per Unit Length and Unit Area of the Knitted Fabric, cm^−1^		Mean Loop Length, *l_m_*, mm	Tightness Factor *TF,* tex^1/2^/mm	Mass per Unit Area of the Knitted Fabric, g/m^2^
					Courses, Pv	Wales, Ph			
1	I—inner II—middle III—outer	PLA spun yarns, 19.7 PLA textured yarns, 16.7/36f HA spun yarns (raw white + dyed) 19.2	E2, E5 E3, E6 E1, E4	35 29 36	11	13	4.337	1.719	345
2	I—inner II—middle III—outer	PLA spun yarns, 19.7 PLA textured yarns, 16.7/36f HA spun yarns (raw white) 27.8 + Hemp (HA) spun yarns (dyed) 19.2	E2, E5 E3, E6 E1, E4	32 28 40	11	13	4.493	1.745	380
3	I—inner II—middle III—outer	PLA spun yarns, 19.7 PLA textured yarns, 16.7/36f HA spun yarns (dyed) 27.8	E2, E5 E3, E6 E1, E4	29 27 44	11	13	4.573	1.752	420
4	I—inner II—middle III—outer	PLA spun yarns, 19.7 PLA textured yarns, 16.7/36f Twisted HA spun yarns (raw white + dyed), 19.2 x2	E2, E5 E3, E6 E1, E4	24 21 55	12	12	4.499	1.922	485

**Table 2:** Summary data of moisture management properties of fabrics investigated.

Fabric No.	Wetting Time (Top), WT_T_, s Rate-Index	Wetting Time (Bottom), WT_B_, s Rate-Index	Absorption Rate (Top), AR_T_, %/s Rate-Index	Absorption Rate (Bottom), AR_B_, %/s Rate-Index	Max Wetted Radius (Top), MWR_T_, mm Rate-Index	Max Wetted Radius (Bottom), MWR_B_, mm Rate-Index	Spreading Speed (Top), SS_T_, mm/s Rate-Index	Spreading Speed (Bottom), SS_B_, mm/s Rate-Index	Accumulative One-Way Transport Capability, AOTC, %, Rate-Index	Overall Moisture Management Capacity, OMMC Rate-Index
1	12.66 Medium—3	7.00 Medium—3	24.76 Slow—2	49.14 Medium—3	10 Small—2	16.67 Medium—3	0.83 Very slow—1	1.90 Slow—2	267.08 Very good—4	0.5359 Good—3
2	16.11 Medium—3	5.20 Medium—3	10.30 Slow—2	23.03 Slow—2	6.67 No wetting—1	15 Medium—3	0.44 Very slow—1	2.01 Medium—3	385.29 Very good—4	0.6026 Very good—4
3	7.18 Medium—3	2.94 Very fast—5	22.68 Slow—2	40.79 Medium—3	10 Small—2	16.67 Medium—3	1.34 Slow—2	3.08 Fast—4	275.79 Very good—4	0.6210 Very good—4
4	6.94 Medium—3	2.69 Very fast—5	23.5 8 Slow—2	45.97 Medium—3	11.67 Small—2	16.67 Medium—3	1.66 Slow—2	3.96 Fast—4	306.75 Very good—4	0.7175 Very good—4

**Table 3:** The results of statistical processing of experimental data for each MMT measurement of knitted fabric samples.

Fabric No.		WT_T_, s	WT_B_, s	AR_T_, %/s	AR_B_, %/s	MWR_T_, mm	MWR_B_, mm	SS_T_, mm/s	SS_B_, mm/s	AOTI, %	OMMC
1	average	12.66	7.00	24.76	49.14	10	16.67	0.83	1.90	267.08	0.5359
	max	15.09	7.88	37.35	53.87	10	20	1.01	2.21	339.25	0.6301
	min	9.75	5.72	15.61	43.96	10	15	0.70	1.58	187.62	0.4588
	SD	2.70	1.14	11.27	4.97	0	2.89	0.16	0.32	76.07	0.0862
	CV	0.21	0.16	0.46	0.10	0	0.17	0.19	0.16	0.28	0.16
	CI	±3.06	±1.29	±12.75	±5.63	±0	±3.27	±0.18	±0.36	±86.09	±0.0976
2	average	16.11	5.20	10.30	23.03	6.67	15	0.44	2.01	385.29	0.6026
	max	18.05	7.64	10.83	25.14	10	15	0.73	2.37	403.65	0.6529
	min	14.39	3.44	9.73	20.07	5	15	0.27	1.44	361.69	0.5218
	SD	1.84	2.18	0.55	2,64	2.89	0	0.25	0.50	21.47	0.0706
	CV	0.11	0.42	0.05	0.11	0.43	0	0.58	0.25	0.06	0.1172
	CI	±2.08	±2.47	±0.63	±2.99	±3.27	±0	±0.29	±0.57	±24.29	±0.0799
3	average	7.18	2.94	22.68	40.79	10	16.67	1.34	3.08	275.79	0.621
	max	8.47	3.06	28.38	42.38	10	20	1.47	3.25	335.82	0.7058
	min	6.09	2.73	19.48	38.51	10	15	1.21	2.87	237.73	0.5551
	SD	1.20	0.18	4.95	2.03	0	2.89	0.13	0.19	52.61	0.0771
	CV	0.17	0.06	0.22	0.05	0	0.17	0.10	0.06	0.19	0.12
	CI	±1.33	±0.20	±5.61	±2.29	±0	±3.27	±0.15	±0.21	±59.53	±0.0872
4	average	6.94	2.69	23.58	45.97	11,677	16.67	1.66	3.96	306.75	0.7175
	max	8.16	2.83	30.00	48.54	15	20	2.44	4.92	321.40	0.7697
	min	5.16	3.63	16.90	41.71	10	15	1.16	3.09	284.50	0.6672
	SD	1.58	0.12	6.55	3.72	2.89	2.89	0.68	0.92	19.59	0.05
	CV	0.23	0.04	0.28	0.08	0.25	0.17	0.41	0.23	0.06	0.0715
	CI	±1.79	±0.13	±7.42	±4.21	±3.27	±3.27	±0.77	±1.04	±22.17	±0.06

**Table 4:** Results of statistical processing of experimental data for each WVP, air permeability and R_ct_ measurement of knitted fabric samples.

Fabric No.		WVP, mg/m^2^ s	Air Permeability, mm/s	R_ct,_ m^2^K/W
1	average	58.57	819.1	0.024
	max	62.78	875.6	0.025
	min	54.36	762.6	0.023
	SD	4.21	56.5	0.001
	CV	0.072	0.068	0.042
	CI	±4.76	±63.94	±0.001
2	average	56.83	796.4	0.034
	max	61.06	851.6	0.035
	min	52.60	741.2	0.033
	SD	4.23	55.2	0.029
	CV	0.074	0.069	0.029
	CI	±4.79	±62.47	±0.001
3	average	56.04	686.2	0.038
	max	60.25	735.9	0.039
	min	51.83	636.5	0.037
	SD	4.21	49.7	0.001
	CV	0.075	0.072	0.026
	CI	±4.764	±56.24	±0.001
4	average	50.39	571.7	0.040
	max	54.20	611.8	0.042
	min	46.58	531.6	0.038
	SD	3.81	40.1	0.002
	CV	0.076	0.070	0.050
	CI	±4.31	±45.38	±0.002

**Table 5:** Characteristics of thermoregulatory properties and performance levels of moisture management knitted fabrics according to summary grading.

Fabric No.	Moisture Management Parameters		Water Vapour Permeability, WVP, mg/m^2^ s	Thermal Resistance (R_ct_)_,_ m^2^K/W	Air Permeability, mm/s	Performance Level (CEN/TR 16422)			
	One-Way Transport Capability, AOTC, %, Rate-Index	Overall Moisture Management Capability (OMMC)				For Skin Contact Materials		For Second (Intermediate) Layer Materials	
						According to R_ct_ Value	According to OMMC and Their Index Value	According to R_ct_ Value	According to OMMC and Their Index Value
1	267.08 Very good—index 4	0.5359 Good—index 3	58.57	0.024	819.1	Warm climate—B (good); Cold climate C (medium)	Warm/cold climate—B (good)	Warm climate—B (good); Cold climate C (medium)	Warm/cold climate—A (very good)
2	385.29 Very good—index 4	0.6026 Very good—index 4	56.83	0.027	796.4	Warm/cold climate—C (medium)	Warm/cold climate—A (very good)	Warm climate—B (good); Cold climate C (medium)	Warm/cold climate—A (very good)
3	275.79 Very good—index 4	0.621 Very good—index 4	56.04	0.038	586.2	Warm/cold climate—C (medium)	Warm/cold climate—A (very good)	Warm/cold climate—C (medium)	Warm/cold climate—A (very good)
4	306.75 Very good index—4	0.7175 Very good—index 4	50.39	0.040	571.7	Warm/cold climate—C (medium)	Warm/cold climate—A (very good)	Warm/cold climate—C (medium)	Warm/cold climate—A (very good)

## Data Availability

The original contributions presented in this study are included in this article; further inquiries can be directed to the corresponding author.
